# Titanium Dioxide Nanoparticles Alter the Cellular Phosphoproteome in A549 Cells

**DOI:** 10.3390/nano10020185

**Published:** 2020-01-21

**Authors:** Mathilde Biola-Clier, Jean-Charles Gaillard, Thierry Rabilloud, Jean Armengaud, Marie Carriere

**Affiliations:** 1 Univ. Grenoble-Alpes, IRIG, SyMMES, CIBEST, F-38000 Grenoble, France; mathilde.clier@gmail.com; 2 Laboratoire Innovations technologiques pour la Détection et le Diagnostic (Li2D), Service de Pharmacologie et Immunoanalyse (SPI), CEA, INRA, F-30207 Bagnols-sur-Cèze, France; jean-charles.gaillard@cea.fr; 3 Chemistry and Biology of Metals, Univ. Grenoble Alpes, CNRS UMR5249, CEA, IRIG-DIESE-LCBM-ProMD, F-38054 Grenoble, France; thierry.rabilloud@cnrs.fr

**Keywords:** omics, lung, inhalation, nanoparticle, TiO_2_, proteomics, phosphoproteomics

## Abstract

TiO_2_ nanoparticles (NPs) are one of the most produced NPs worldwide and are used in many consumer products. Their impact on human health, especially through inhalation, has been studied for more than two decades. TiO_2_ is known for its strong affinity towards phosphates, and consequently interaction with cellular phosphates may be one of the mechanisms driving its toxicity. In the present study, we used a phosphoproteomics approach to document the interaction of TiO_2_-NP with phosphoproteins from A549 human pulmonary alveolar epithelial cells. Cells were exposed to 21 nm anatase/rutile TiO_2_-NPs, then their phosphopeptides were extracted and analyzed using shotgun proteomics. By comparing the phosphoprotein content, phosphorylation status and phosphorylation sites of exposed cells with that of control cells, our results show that by affecting the phosphoproteome, TiO_2_-NPs affect cellular processes such as apoptosis, linked with cell cycle and the DNA damage response, TP53 being central to these pathways. Other pathways including inflammation and molecular transport are also affected. These molecular mechanisms of TiO_2_-NP toxicity have been reported previously, our study shows for the first time that they may derive from phosphoproteome modulation, which could be one of their upstream regulators.

## 1. Introduction

Titanium dioxide (TiO_2_) is widely used in daily products as a white pigment, for example, as a food additive and in paints [1]. It is also used in cosmetics as a UV filter [1]. The annual production of nanosized TiO_2_ ranges between 3800 and 7800 tons in the US. With such a large production, TiO_2_-NPs could be present in 9% of nanomaterial-containing products [2].

The impact of TiO_2_-NPs on the lung has been the subject of intense research since inhalation is considered as being the first and most problematic route of occupational exposure. In vivo, exposure to TiO_2_-NP either via intratracheal instillation or inhalation causes pulmonary inflammation, fibrosis and emphysema-like response [1,3,4,5]. TiO_2_ is classified as possibly carcinogenic (2B group) via inhalation by the International Agency for Research on Cancer (IARC) [6]. In vitro, TiO_2_-NPs are endocytosed in a plethora of cell models [7]. When internalized in cells, TiO_2_ accumulates in endosomes and is transferred to lysosomes. Although there is no evidence of its accumulation in mitochondria, TiO_2_ induces oxidative stress in exposed cells by increasing the intracellular level of reactive oxygen species (ROS) and impairing the antioxidant cellular response [3,8]. This effect has been shown to be associated with the inhibition of the nuclear factor (erythroid-derived 2)-like 2 (NRF2) in BEAS-2B lung cells [9] and in mice kidneys [10]. TiO_2_ also causes oxidative damage to DNA without accumulating inside cell nucleus, except if the cell has undergone mitosis, one of the stages of which is the rupture of the nuclear envelope [11]. This genotoxicity is concomitant to impairment of the mitotic spindle assembly and function [12], decreased DNA repair activities [13,14] and cell cycle progression [15,16,17,18,19,20], suggesting that TiO_2_-NPs exert indirect primary genotoxicity [11]. Finally, TiO_2_-NPs have been shown to affect the autophagic process [21,22], and this property has been proposed as a possible therapeutic approach to treat cancer [23]. The main driver of these cellular effects is currently unknown and whether multiple factors are involved remains to be established.

Phosphate is essential for cell survival. Protein phosphorylation/dephosphorylation reactions, carried out by kinases and phosphatases, regulate the activity of almost all cellular processes [24,25] TiO_2_ shows high affinity for phosphate groups, due to its ion exchange abilities and ligand exchange behavior towards Lewis bases (for review, see [26]) and for this reason it has long been used to purify phosphoproteins [27,28]. Since TiO_2_-NPs heavily accumulate in cytoplasmic vesicles, they may adsorb phosphoproteins on their surface, sequester them in these vesicles, and consequently interfere with their proper function. Moreover, kinases and phosphatases are phosphoproteins; their sequestration on the surface of TiO_2_-NPs may hinder their role in maintaining the pool of cellular phosphoproteins. Therefore, we hypothesized that the impact of TiO_2_ on protein phosphorylation status could be one upstream mechanism driving its toxicity. This hypothesis is supported by some results showing impact of TiO_2_-NPs on cellular phosphoproteins. For instance, exposure of BEAS-2B cells to TiO_2_-NPs increases the phosphorylation level of several central kinases, including P38, c-Jun NH_2_- terminal protein kinases (JNKs), mitogen-activated protein kinase/extracellular signal-regulated kinase (ERK1/2), mitogen- and stress-activated protein (MSK1), glycogen synthase kinase (GSK), AMP-activated protein kinase (AMPK), signal transducer and activator of transcription (STAT) and the tyrosine protein-kinase FYN [29].

Consequently, the aim of this study was to characterize the impact of TiO_2_-NPs on protein phosphorylation profile in lung cells. Rather than using a case-by-case method, we used a large-scale approach, i.e., phosphoproteomics. This approach provides an exhaustive view of cellular phosphoproteome alteration in cells exposed to TiO_2_-NPs as compared to unexposed cells. We used a label-free phosphoproteomics approach consisting in enriching cellular phosphopeptides, then identifying and quantifying them by shotgun proteomics. This approach was applied to A549 human lung epithelial alveolar cells, exposed for 24 h to 100 µg/mL of 21 nm anatase/rutile TiO_2_-NP, i.e., 17 µg/cm^2^ equivalent to 0.11 ng/cell. This concentration corresponds to the alveolar deposition of TiO_2_-NP after inhalation exposure to 1 mg/m^3^ for a whole working lifetime [30]. Therefore, it can be considered as a worst-case exposure scenario. This exposure condition leads to moderate cell mortality, as inferred from our previous studies [14,31].

## 2. Materials and Methods

### 2.1. Chemicals and Nanoparticles

Unless otherwise indicated, chemicals were purchased from Sigma-Aldrich (Saint-Quentin Fallavier, France) and were >99% pure. NM105 TiO_2_-NPs were supplied by the Joint Research Center of the European Commission (JRC, Ispra, Italy). These NPs exhibit a spherical, ellipsoidal, and cuboidal structure, are 86% anatase and 14% rutile. Their diameter is 21 nm and their specific surface area 46 m^2^/g [32]. NPs were dispersed in ultrapure sterile water, by sonicating them in water for 30 min at 4 °C, using a high energy sonicator in pulsed mode (1 s on/1 s off), using an Autotune 750 W sonicator from Fisher Bioblock Scientific (Rungis, France) operated at 30% of amplitude, as previously described [14,31]. According to the calorimetric procedure, this corresponds to 19.82 W [33,34]. Suspensions were diluted in serum-free cell culture medium immediately before cell exposure. Their hydrodynamic diameter was 70 ± 20 nm with a 0.18 ± 0.04 polydispersity index (PDI) in water; it shifted to 720 ± 20 nm (PDI: 0.50 ± 0.02) after dilution in the exposure medium (mean ± standard deviation, n = 3, not shown).

### 2.2. Cell Culture and Exposure Conditions

This study was performed on A549 human epithelial alveolar cells (A549, ATCC CCL-185), grown in DMEM as previously [14]. The use of this cell line in toxicity studies has been criticized because it exhibits a constitutively active NRF2 [35]. However, we chose to use it in the present study because to our knowledge it is the only available cell line of human alveolar origin and NPs have been shown to deposit in this region of the lung due to their small size [36]. They were exposed, 3 days after seeding, to 100 µg/mL of TiO_2_-NPs diluted in serum-free cell culture medium, with three biological replicates per condition (n = 3), then rinsed three times with PBS and harvested using trypsin. This concentration corresponds to 17 µg TiO_2_/cm^2^, i.e., 0.11 ng TiO_2_/cell. At this concentration, these NPs cause approximately 25% of cell viability loss (as shown in [31], where these NPs are referred to as “TiO_2_-Degussa” and in [13]), but significantly increases intracellular reactive oxygen species level, causes DNA damage and significantly increases the DNA repair activities in A549 cells (as shown in [14], in this article these NPs are referred to as “A25”, and in [13]). Cell exposure was performed in the dark in order to avoid any photocatalytic effect of TiO_2_-NPs.

### 2.3. Phosphoproteome Sample Preparation and Trypsin Proteolysis

The phosphoproteomics experiment was performed with 1.2 × 10^7^ cells per replicate, which were sampled using trypsin and flash frozen in liquid nitrogen. Samples were stored at −80 °C until analysis. The samples were melted on ice in a lysis buffer consisting in 50 mM Tris/HCl, pH 8.2, 8 M urea, 75 mM NaCl and 2X phosphatase inhibitor cocktail (Thermo Scientific, Illkirch, France, 88667). They were then sonicated three times using a Hielsher (Teltow, Germany) UP50H probe sonicator, operated in pulse mode (20 s at 0.4 s on/0.4 s off) at 40% amplitude, with a pause of 1 min between each cycle of sonication. After centrifugating these lysates at 16,000 × rcf for 10 min, 4 °C, the supernatants were stored at −80 °C. After thawing, protein concentration in the samples was measured using Bradford reagent (Interchim, Montluçon, France, CooAssay Standard Protein Assay kit), using the protocol of the supplier. The reduced cysteine residues were alkylated by reaction with iodoacetamide (5 mM), for 15 min at room temperature. After dilution with 100 mM Tris/HCl, pH 8.5, to reach an urea concentration of 1 M, the samples were digested overnight at 37 °C using 1 mg/mL trypsin prepared in a 0.01% aqueous solution of trifluoroacetic acid, so that the final concentration of protein was 2% per sample. After centrifugating for 1 min at 1000 rcf, at room temperature, the supernatants were stored at −80 °C. SDS-PAGE followed by Coomassie blue staining was used to evaluate the extent of proteolysis.

### 2.4. Phosphopeptide Enrichment by Chromatography

Digested samples were desalted using Harvard Apparatus (Holliston, MA, USA) Macro Spin C18 Columns, using the protocol of the supplier, consisting in (i) activation of the resin in 500 µL of 80% acetonitrile, 20% water, repeated three times, (ii) equilibration of the columns by rinsing with 500 µL 0.5% trifluoroacetic acid, three times. 250 µg of digested peptides was loaded onto columns, which were then centrifuged at room temperature for 30 s at 2000 rcf. The filtrates were then collected and loaded again onto the columns. At the end of this procedure, the resins were rinsed three times with 0.5% trifluoroacetic acid and peptides were eluted in 100 µL of 1:1 acetonitrile/water, 0.1% TFA. These desalted peptides were freeze-dried and stored at −80 °C.

**Figure 1:**
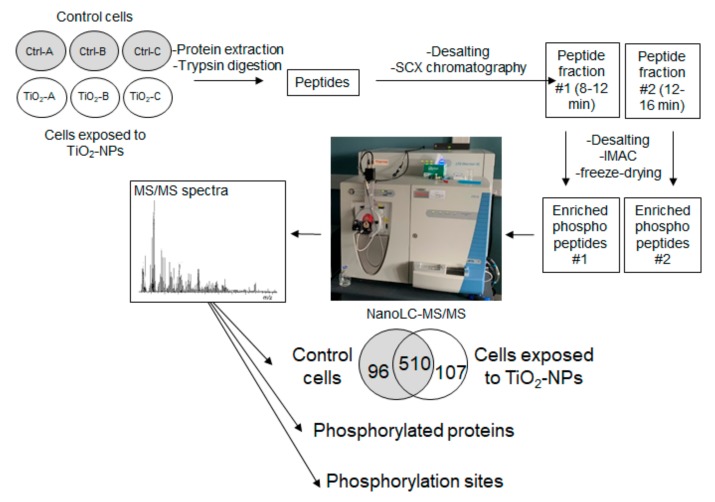
Experimental strategy. The phosphoproteomic analysis was performed on control (Ctrl) and TiO_2_-NP-exposed cells (TiO_2_), with three biological replicates per condition (A, B and C). SCX: strong cation exchange (chromatography); IMAC: immobilized metal affinity chromatography; NanoLC-MS/MS: nano liquid chromatography coupled to tandem mass spectrometry.

As described in Figure 1, the samples were then subjected to two consecutive chromatographies. The first one was a strong cation exchange chromatography (SCX), which was processed as previously described [37], using a polysulfoethyl ATM column (PolyLC, Columbia, MD, USA, 3.2-mm inner diameter, 200 mm length, 5-µm particle size, 200 Å pore size) operated at a flow rate of 400 µL/min on a 1100 Series reverse-phase high performance liquid chromatography (HPLC) system equipped with a G1315B diode array detector, a G1322A degasser and a G1311A quaternary pump, as well as a G1329A autosampler, and sample cooler unit from Agilent (Les Ulis, France) [38]. Samples (1 mg of peptide) were diluted in 65 µL of buffer A (7 mM KH_2_PO_4_, pH 2.65 and 30% acetonitrile (vol./vol.)) and 60 µL was injected in the column. The stepwise elution was performed with three buffers: buffer A, buffer B (7 mM KH_2_PO_4_, 350 mM KCl, pH 2.65, 30% acetonitrile (vol./vol.)), buffer C (50 mM K_2_HPO_4_, 500 mM NaCl, pH 7.5) and ultrapure water. Buffer A was applied to the column from time 0 to min 34. Then we applied 75% buffer A and 25% buffer B for one min, then buffer B from min 36 to 41, then ultrapure water from min 42 to 48. Buffer C was then applied from min 49 to 60, followed by ultrapure water from min 61 to 67. Finally, Buffer A was applied from min 68 to 120. Phosphopeptide samples were collected as two separate fractions of 4 min-elution, between min 8 and min 16, as described previously [37]. These two separate fractions were freeze-dried in order to reach a final volume of 150 µL, to which was added 350 µL of 0.1% TFA. The samples were desalted as previously. The second chromatography was used to enrich phosphopeptides, and consisted in immobilized metal affinity chromatography (IMAC). The resin was the PhosSelect Iron Affinity Gel resin (Sigma-Aldrich, Saint-Quentin Fallavier, France, P9740), to which samples were bound as previously described [37]. Peptides were then desalted as described above, except that samples were rinsed with 200 µL instead of 500 µL. To do so, the resin was loaded on activated and equilibrated Harvard Apparatus (Holliston, MA, USA) Micro Spin C18 columns. Therefore, we obtained a two-layered resin, which was rinsed twice with 100 µL of 40% acetonitrile, 60% 25 mM formic acid (IMAC binding buffer) and once with 200 µL of C18 wash solution. Phosphopeptides were eluted from the PhosSelect resin and retained on the underlying C18 resin by adding three times 100 µL of 500 mM K_2_HPO_4_, pH 7 (IMAC elution buffer). The resin was rinsed four times with 200 µL of C18 wash solution and phosphopeptides were eluted twice using 50 µL of C18 elution solution. These two eluted fractions were pooled. Samples were then freeze-dried and stored at −80 °C.

### 2.5. Tandem Mass Spectrometry

Phosphopeptides (10 µL) were loaded on the LTQ Orbitrap XL hybrid nano-liquid chromatography coupled to tandem mass spectrometry (nanoLC-MS/MS) system (ThermoFisher Scientific, Les Ulis, France) coupled to Ultimate 3000 LC system (Dionex-ThermoFisher Scientific, Les Ulis, France), after dissolution in 30 µL of 0.1% TFA. The analysis conditions were as described by Dedieu et al. [39]. Full-scan mass spectra were measured from *m*/*z* 300 to 1800. The mass spectrometer was operated in data-dependent mode using a TOP3 strategy consisting in a scan cycle initiated with a full scan of high mass accuracy in the Orbitrap (30,000 resolution; internal calibration), followed in parallel by MS/MS normal mode scans in the linear ion trap on the three most abundant precursor ions. The parameters were set as follows: minimum signal required: 15,000; possible charge states: 2+ and 3+; dynamic exclusion of previously-selected ions with 60 sec exclusion duration. The previously described Multi Stage Activation mode (pseudo-MS3) [40] was activated with a neutral lost mass list including 32.6590 (phosphate, triple positive charges) and 48.9890 (phosphate, double positive charges). The lock mass option on the LTQ Orbitrap XL mass spectrometer was enabled in MS mode and the polydimethylcyclosiloxane ions generated in the electrospray process from ambient air (protonated [(CH_3_)_2_SiO)]_6_ with *m*/*z* at 445.12002) were used for internal recalibration in real time.

### 2.6. MS/MS Spectra Interpretation, Statistics, and Data Mining

Peak lists were generated with the MASCOT DAEMON software (Matrix Science, London, UK, version 2.3.2) using the extract_msn.exe data import filter from the Xcalibur FT package (version 2.0.7) (ThermoFisher Scientific, Les Ulis, France). The filtering options were, as previously [41]: minimum mass: 400, maximum mass: 5000, grouping tolerance: 0, intermediate scans: 0, minimum peaks: 10, extract MSn: 2 and threshold: 1000. Peptides were assigned from MS/MS spectra according to the SwissProt database with the MASCOT 2.3.02 software (Matrix Science, London, UK), by searching the database with the following parameters: Mammalia taxonomy (65,476 sequences), SwissProt_2012_02, maximum number of miss-cleavages: 2; mass tolerances: 5 ppm (parent ion) and 0.5 Da (MS/MS); carbamidomethylated Cys: fixed modification; and oxidized Met and phosphorylated Ser, Thr and Tyr residues: variable modification. MASCOT results were analyzed using IRMa 1.30.4 [32] software, which filters out peptides with a p-value of less than 0.01 and a rank set to 1. Fold-changes represent the ratio of values obtained in samples exposed to TiO_2_ and samples from control cells, based on spectral counts after standard normalisation. PatternLab software was used for normalization and statistical significance assessment [42]. The cut-off values to consider results as statistically significant was *p* < 0.05 and fold-change > 1.5. Gene ontology and pathway analyses were performed using Database for Annotation, Visualization and Integrated Discovery (David) [43,44] and Ingenuity^®^ Pathway Analysis (IPA^®^—23814503, QIAGEN, Courtaboeuf, France). Enriched pathways were considered statistically significant when *p* < 0.05.

### 2.7. Cell Cycle Analysis

Cells were exposed to 100 µg/mL of TiO_2_-NPs, then rinsed three times with PBS containing 2 mM of EDTA. Then they were fixed for 30 min in 70% ice-cold ethanol and diluted in PBS-EDTA (2 mM). This fixative solution was removed and cells were suspended in 25 µg/mL propidium iodide prepared in PBS-EDTA (2 mM) to which was added 25 µg/mL RNase A. Samples were analyzed by flow cytometry using a FACS Calibur (Becton Dickinson, Rungis, France). This experiment was repeated three times independently, with four replicates per experiment.

### 2.8. Electron Microscopy

Cells exposed to 100 µg/mL TiO_2_-NPs for 24 h were washed three times with phosphate saline buffer, fixed with 2.5% glutaraldehyde and post-fixed with OsO_4_. They were then dehydrated by immersion in solutions of ethanol with increasing concentration and embedded in Epon. Ultrathin sections were prepared by ultramicrotomy and counterstained with uranyl acetate and lead citrate. These samples were observed using a CM12 Philips electron microscope, operating at 80 kV.

## 3. Results and Discussion

Cell exposure conditions were chosen based on results that we previously obtained, showing mild cytotoxicity of TiO_2_-NPs to A549 cells, i.e., less than 25% of cell mortality in exposed cells according to the MTT assay. Moreover, we previously showed that this exposure condition leads to a significant elevation of intracellular reactive oxygen species level, suggesting oxidative stress. We also showed that it causes significant DNA damage, as assessed via the comet assay and quantification of 8-oxo-dGuo using high performance liquid chromatography coupled to tandem mass spectrometry (HPLC-MS/MS), as well as elevation of DNA repair capacities in exposed cells [13,14]. The same conclusions were obtained on BEAS-2B cells, which is a normal bronchial cells [13].

### 3.1. Phosphoproteome Analysis

Phosphoproteomes of control cells and cells exposed to TiO_2_-NPs were analyzed after enrichment by high-resolution tandem mass spectrometry [37]. As shown in Figure 1, phosphopeptides in both non-exposed and exposed cells were enriched for each of the three biological replicates, resulting in two separate fractions (#1 and #2) per sample. These two samples were analyzed separately over a 90-min gradient by nanoLC-MS/MS with a high-resolution mass spectrometer, and the results were merged (Supplementary Table S1).

Among the recorded spectra for the three biological replicates 10,472 and 11,754 were assigned to human peptides in control cells and cells exposed to TiO_2_-NPs, respectively, corresponding respectively to 1310 and 1283 unique peptides (Supplementary Table S1). Among them, 88–90% were phosphorylated: they mainly carried one phosphorylation (66–70%), but sometimes two (18–24%) and more rarely three (0.2%). This corresponds to 1606 unique phosphorylation sites (P-sites), which are listed in Supplementary Table S2. These peptides belong to 644 and 649 proteins in control cells and cells exposed to TiO_2_-NPs, respectively, 96% and 93% of them being phosphorylated, i.e., 606 phosphoproteins in control cells and 617 phosphoproteins in cells exposed to TiO_2_-NPs that are reported in Table S3. Among these proteins, 510 were detected in both control cells and cells exposed to TiO_2_-NPs (Supplementary Table S3, Figure 1).

### 3.2. Comparison of Phosphorylation Levels in Control Cells and Cells Exposed to TiO_2_-NPs

**Figure 2:**
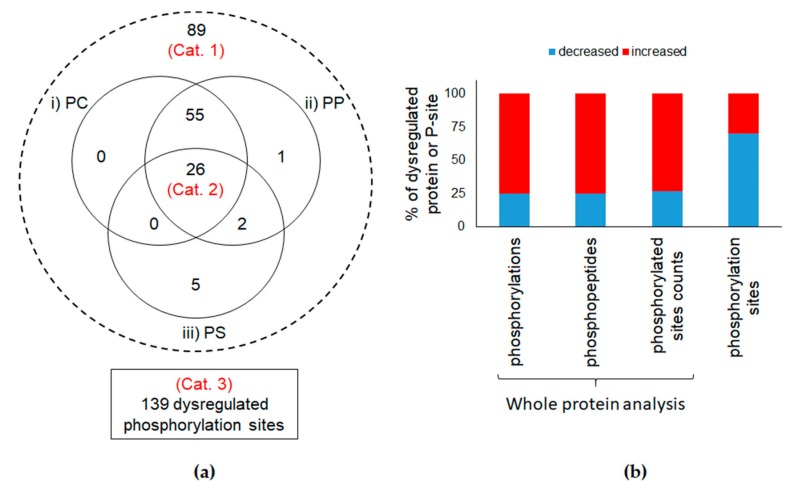
Comparison of different phosphorylation levels. (**a**) Distribution of the number of proteins included in the different study groups. Whole-protein analysis groups (Cat. 1 and 2) and phosphorylation sites (Cat. 3); (**b**) Distribution of up- (red) or down-regulated (blue) (fold-change ≥ 1.5, *p*-value < 0.05) phosphorylation levels in terms of whole protein indicators (phosphorylation, phosphopeptide and phosphorylated site counts) or phosphorylation level.

We compared the protein phosphorylation level in control cells and in cells exposed to TiO_2_-NPs using three criteria. First, we compared the phosphorylation counts, i.e., the numbers of phosphorylated residues detected on all the peptide sequences taking into account the Peptide-to-Spectrum Matches. The second criterion was the phosphopeptide count, i.e., the number of spectra of phosphopeptides detected in the samples. The third criterion was the phosphorylated sites count, i.e., the number of unique phosphorylated sites on the detected peptides (see Figure S1 for a schematic representation). Using these three criteria, when analyzing the list of phosphopeptides detected in the samples, normalized with respect to individual protein molecular weights, the overall phosphorylation level did not statistically differ in CTL cells, compared to cells exposed to TiO_2_-NPs (Figure S2). When using these three criteria to analyze the phosphorylation level of each individual phosphoprotein, we identified significant differences between control cells and cells exposed to TiO_2_-NPs. The fact that these two complementary analyses led to opposite phosphorylation trends suggests that many phosphorylatable sites (P-sites) showed different phosphorylation level rather than a strong modification of phosphorylation level of a few P-sites (here, phosphorylatable sites are considered as those that have been identified phosphorylated at least once in a biological sample in the experiment). As reported in Figure 2, out of the 510 phosphoproteins identified in both control and TiO_2_-exposed cells, 89 proteins (17.5%) showed different phosphorylation levels in cells exposed to TiO_2_-NPs as compared to control cells according to at least one of the three criteria (Category 1). Twenty-six proteins showed different phosphorylation levels according to all three criteria (Category 2). Most of these phosphoproteins showed increased phosphorylation levels in cells exposed to TiO_2_-NPs, compared to control cells (Figure 2).

When using the same approach on individual P-sites, out of the 1606 detected P-sites, 139 showed different phosphorylation levels in control and TiO_2_-exposed cells. These 139 P-sites belonged to 111 phosphoproteins (Category 3). Contrary to what was found when analyzing protein phosphorylation level, 70% of P-sites were less phosphorylated in TiO_2_-NP-exposed cells than in control cells. Therefore, the overall protein phosphorylation level is not affected in cells exposed to TiO_2_-NPs. Considering the strong affinity of TiO_2_ towards phosphate groups, and the intense accumulation of these TiO_2_-NPs in A549 cells (see [31], where these TiO_2_-NPs are referred to as TiO_2_-Degussa), a much more intense dysregulation of the overall protein phosphorylation levels was expected. Our results suggest that the impact of TiO_2_-NPs on the cellular phosphoproteome is much more finely tuned. This could be explained by the cellular distribution of TiO_2_-NPs in A549 cells, which shows preferential location in endosomes and/or lysosomes [31]. Sequestration in such compartments would limit the contact of TiO_2_ with phosphoproteins.

### 3.3. Functional Analysis

#### 3.3.1. Protein Ontology

The list of phosphocounts, phosphopeptides and phosphosites showing modulated phosphorylation level (*p* < 0.05) was analyzed via protein ontology, using David and Ingenuity^®^ Pathway Analysis (IPA, Qiagen, Courtaboeuf, France), followed by data mining using the Uniprot protein annotation. The whole human proteome was used as reference group. Since both David and IPA analyses, based on the lists of individual P-sites, phosphocounts or phosphopeptides, highlighted the same cellular functions and processes, we present here the results obtained using David and the analysis performed on the list of phosphopeptides, showing a false discovery rate (FDR) lower than 20% (Table 1). All GO terms identified on the list of phosphopeptides are reported in Table S4.

The list of GO terms obtained when analyzing phosphocounts (cluster analysis) and P-sites are reported in Supplementary Tables S5 and S6, respectively. The GO terms obtained via IPA, in the pathways “Diseases and Biofunctions”, for proteins listed in category 1, 2, and 3 are reported in Supplementary Tables S7–S9, respectively. For most of these proteins, the link between the phosphorylation status and their function is not established. Therefore, here, we report the overall function of these phosphoproteins, and if available, the role played by phosphorylation in their function.

Among the GO terms identified by David, “apoptosis” (FDR 6.95%) and “negative regulation of extrinsic apoptotic signaling pathway” (FDR 18%) attracted our attention, since apoptosis has already been described as being one of the cellular processes induced by TiO_2_-NPs [1]. The proteins identified here and involved in these pathways are EPHA2, BAG6, ACIN1, HTT, BAG3, MAP1S, SQSTM1, TPX2, CTTN, SON, KRT18, ADAR, and LYRIC. EPHA2 participates in UV-induced apoptosis [45] and ACIN1 induces chromatin condensation [46]. BAG3 exhibits anti-apoptotic activity when bound to BCL-2 [47]. TPX2 participates in the assembly of microtubules during apoptosis [48]. Some of these proteins also play roles in the autophagic process, which is also known as being dysregulated in cells exposed to TiO_2_-NPs [22,49,50]. For instance, MAP1S activates autophagy and consequently reduces cell propensity to undergo apoptosis [51]. HTT is implicated in autophagic vesicles formation [52] and it is phosphorylated in response to DNA damage; its phosphorylation is linked with its toxicity [53]. SQSTM1, when linked to p62, constitutes a selective autophagy receptor that directs ubiquitinated substrates to degradation [54], while SPP1 regulates ER stress-induced autophagy [55] and RB1 is also involved in autophagy [51]. In line with this observation, we previously showed that repeated exposure of A549 cells for 2 months to the same TiO_2_-NPs as in the present study leads to significant accumulation of TiO_2_-NPs in autophagic vesicles and increased LC3II/LC3I ratio, suggesting alteration of the autophagic process [49]. This alteration would result from modulation of the phosphorylation of these proteins. Moreover, some of these proteins play roles in the DNA damage response, which is activated in TiO_2_-NP-exposed cells, particularly via impairment of the DNA repair as previously demonstrated in A549 cells exposed to the same TiO_2_-NPs and in the same conditions as in the present study [13,14] and via modulation of cell cycle progression [15,16,17,18,19,20]. This is the case of BAG6, which is involved in DNA-damage induced apoptosis by binding EP300, itself involved in the regulation of TP53 transcriptional activity [56]. Moreover, phosphorylated ACIN1 modulates the expression of cyclin A1 that is involved in the control of cell cycle progression, particularly in the transitions between G1 and S phase and between G2 and M phase [57]. SON is involved in splicing of many DNA-repair transcripts, and is required for the progression of the cell cycle [58]. In addition, ITGAV is involved in TGF-β1 activation [59], while RB1 regulates the entry into cell division. It is either active or inactive depending on its phosphorylation status. Depending on its phosphorylation level, it is also involved in TGF-β1-induced apoptosis [60]. Moreover, NDRG1 shows different phosphorylation level in cells with active cell cycle or with blocked cell cycle [61,62]. Its phosphorylation level controls its nuclear localization and its role in cell division, DNA repair and proteasomal degradation [62]. Analysis of the cell cycle in A549 cells exposed to 100 µg/mL of TiO_2_-NPs for 24 h shows a mild but statistically significant reduction of the proportion of cells in the G1 phase, as compared to unexposed cells (Supplementary Figure S3). Moreover, after repeated exposure of A549 cells to the same TiO_2_-NPs as in the present study, we previously observed decreased cell proliferation and perturbation of the cell cycle [49,63]. Moreover, a significant increase of the proportion of cells in the sub-G1 phase is observed, implying that some cells undergo apoptosis (Supplementary Figure S3). This suggests that this effect on the cell cycle, visible at the scale of the phosphoproteome, translates into a phenotypic effect, which is revealed after acute and prolonged exposure.

**Figure 3:**
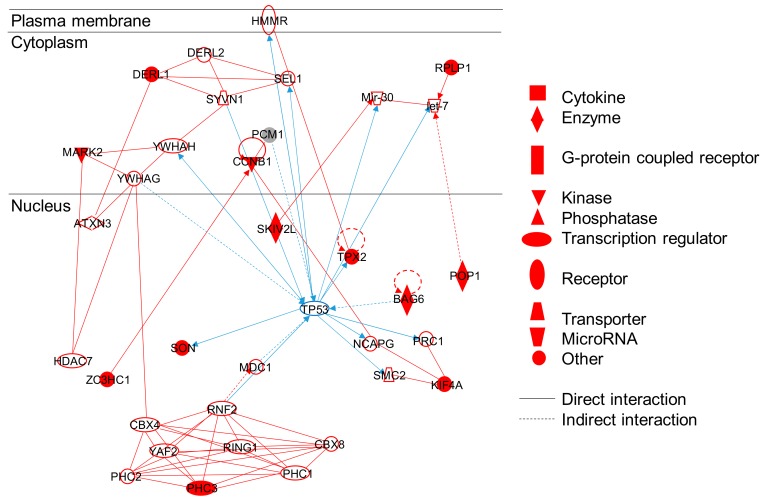
IPA^®^ protein network illustrating the central role of p53 in TiO_2_-NP impact. This IPA^®^ protein network illustrates molecular orchestration of cellular development, movement, and morphology. Proteins depicted in red have been detected in our dataset, while non-colored proteins were not detected. Two genes products showing binding are represented by a line, and an arrow is indicated when a gene product acts on another one. The blue lines highlight the connections to TP53.

Our analysis highlights the central role of TP53 in these processes, as illustrated by a network identified using IPA (Figure 3). This network focuses on cellular assembly and organization, DNA replication, recombination and repair, and cancer pathways. The phosphorylation of TP53 itself is not shown to be affected, but p53 acts as an upstream transcriptional regulator of several proteins having modulated phosphorylation levels (SKIV2L, TPX2, BAG6, SON, KIF4A, POP1, RPLP1, DERL1, MARK2, ZC3HC1, PHC3). We previously showed that repeated exposure of A549 cells to the same TiO_2_-NPs as in the present study, up to two months, leads to increased phosphorylation and acetylation of TP53 [49]. This suggests that the TP53 pathway is activated in A549 cells exposed to these TiO_2_-NPs. Induction of DNA damage and/or apoptosis is known to be related to activation of the TP53 pathway [64,65,66,67,68,69], we show here that these cellular processes are at least partly impacted via dysregulated phosphorylation.

Impaired cell cycle progression induced by TiO_2_-NPs is generally reported as cell accumulation in S or G2-M phases [15,16,17,18,19,20]. It is concomitant with impairment of mitotic progression, formation of multipolar mitotic spindles, abnormal chromosome segregation during anaphase and telophase, with deregulation of the function of PLK1 kinase [12]. The present analysis suggests that dysregulated phosphorylation may be a mechanism through which all these processes are affected.

**Figure 4:**
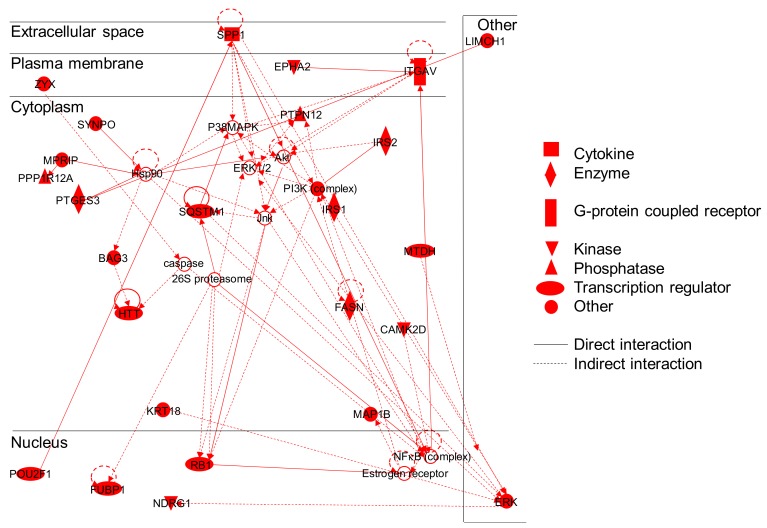
IPA^®^ protein network illustrating the links between proteins involved in TiO_2_-NP impact on the cytoskeleton. Proteins depicted in red have been detected in our analysis, while non-colored proteins were not identified. Two genes products showing binding are represented by a line, and an arrow is indicated when a gene product acts on another one.

Several pathways identified by David-based analysis are related to disturbance of the cytoskeleton, both actin cytoskeleton and microtubules (“Cytoskeleton” FDR 0.05%, “actin cytoskeleton” FDR 0.52%, “actin binding” FDR 2.89%, “microtubule cytoskeleton organization” FDR 6.27%, etc.). Some of these proteins play roles in cell migration, motility, and adhesion. For instance, TLN2 is a component of focal adhesion plates and is involved in cell adhesion in synapses, ZYX is a stabilizer of focal adhesions in muscle cells and synapses [70], and CTTN is involved in cell migration and the formation of metastases [71]. Other proteins rather play roles in cellular structure, such as MAP1B, which is involved in microtubule polymerization and stabilization, in the formation of autophagosomes and in membrane blebbing [72]. Likewise, MPRIP stabilizes actin fibers and plays a role in development of stress fibers [73] and KIF4A is necessary for successful cytokinesis [74]. Moreover, some of these proteins are involved in intracellular trafficking, such as NDRG1, which is involved in trafficking of vesicles and lipids and CTTN, which is involved in protein trafficking and in endocytosis [75]. This impact on the cytoskeleton is illustrated by a second network identified using IPA, which involves 35 proteins and illustrates the impact of TiO_2_-NPs on cellular development, movement and morphology (Figure 4).

**Figure 5:**
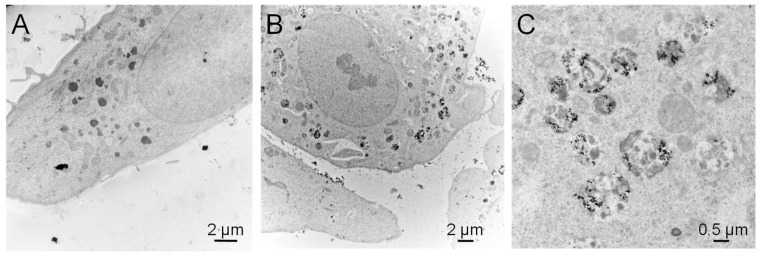
Transmission electron microscopic observation of A549 cells exposed to TiO_2_-NPs. (**A**) Control cells; (**B**,**C**) cells exposed to 100 µg/mL TiO_2_-NPs for 24 h.

Transmission electron microscopic observation of these cells exposed to TiO_2_-NPs showed strong accumulation of NPs inside cells, and localization in cytoplasmic vesicles (Figure 5). This accumulation did not lead to any change in the morphology of cells, and we did not detect the presence of stress fibers (Figure 5).

The impact of TiO_2_-NPs on cytoskeleton integrity highlighted in this phosphoproteome analysis is consistent with the literature [17,76,77,78,79,80,81,82,83,84]. Indeed, TiO_2_-NPs have been shown to impair the expression of genes involved in cytoskeleton maintenance [17,81]. It has also been shown to induce disorganization of microtubules and of the actin network [76,79,80,83]. Moreover, in an acellular study, TiO_2_-NPs were also shown to inhibit tubulin polymerization and change its conformation [77]. Again, we show here that dysregulated phosphorylation of some phosphoproteins may be a mechanism leading to this impact.

In addition, several pathways identified in this gene ontology search are related to RNAs and their processing (“poly(A) RNA binding” FDR 0.03, “alternative splicing” FDR 0.25, “RNA-binding” FDR 19.65), particularly to splicing of pre-mRNA and mRNA (SON, SNRNP200, SRRM1, ADAR, ACIN1). SON is a cofactor for transcript splicing, especially for transcripts related to cell cycle and DNA repair [85], while SNRNP200 is involved in spliceosome assembly and activation and it is a putative helicase [86]. SRRM1 is part of the mRNP granules, which regulate mRNA translation, localization, and recycling [87]. ADAR is involved in the editing of double-stranded RNA [88], which affects mRNA translation. ACIN1 is part of the exon junction complex, which is involved in mRNA processing [89]. One of these proteins is a component of the MRP ribonuclease complex and of ribonuclease P, which is involved in the generation of mature tRNA and (POP1). Finally, one of these proteins is a helicase involved in the biogenesis of 60 S ribosomal subunits (DDX51). This suggests a possible impact of TiO_2_-NPs on translation, which would be mediated by dysregulated phosphorylation of some phosphoproteins. RNA-binding proteins have been reported to bind to SiO_2_ nanoparticles, and consequently to affect translation [90]. The present results suggest that TiO_2_-NPs could also affect translation through modulation of the phosphorylation of proteins involved in RNA processing. This would explain the overall gene expression downregulation that we previously observed in A549 cells exposed to the same TiO_2_-NPs, and in the same exposure conditions [13].

Finally, several proteins are involved in molecular transport (ADAR, CTTN, HTT, NUP214 and SEC61B), in and out of the nucleus in particular. NUP214 is a nucleoporin involved in nuclear export [91] and SEC61B is responsible for the nuclear import of proteins, in particular the epidermal-growth factor receptor (EGFR) [92]. Moreover, SQSTM1 also shows dysregulated phosphorylation level. It has been reported that chromosomal translocation leads to the production of a NUP214-SQSTM1 fusion protein that forms nuclear bodies, which shuttle between the nuclear and cytoplasmic compartments, and may be a form of storage of nuclear transport proteins [93]. Dysregulated phosphorylation levels of NUP214 and SQSTM1 might be the result of dysregulated levels of the single proteins, or of the fusion protein. Modulation of the phosphorylation profile of these proteins might affect the nuclear localization of some key proteins, for instance, some proteins involved in DNA repair, explaining the accumulation of DNA caused by TiO_2_-NPs [11] or impaired DNA repair activities [13,14]. These results are consistent with recent studies, which report that TiO_2_-NPs dysregulate the expression of genes or proteins related to ion transport [81,84], trans-membrane transport [94,95], and molecular transport inside the cell [29,96]. 

#### 3.3.2. Biological Activity of Dysregulated Phosphorylation Sites

We then analyzed the literature relative to the function of the 139 dysregulated P-sites, which would explain some of the impacted biological activities. The biological function of only 14 P-sites is known (Table 2). Some kinases responsible for the phosphorylation of these P-sites have been reported, while phosphatases are known only for two of them, namely CTTN^421^ and CARHSP^141^, which are dephosphorylated by PTP1B and PP2A, respectively [78,97]. Again, the two main functions that are impacted via these dysregulated P-sites are cell cycle regulation and cytoskeleton organization. AKAP^1151^, ENSA^67^, ERF^526^, FLNA^1084^, KIF4A^801^, MISP^397^ and RB^1826^ have been shown to regulate the cell cycle [98,99,100,101,102,103,104], while AKAP^1151^, CTTN^421^, FLNA^1084^, HSPB^115^ and VIM^5^6 have been reported to be involved in cytoskeleton organization [97,98,103,105,106,107,108,109].

Other functions are identified through this approach, i.e., the oxidative stress response (HSPB^115^ and CARHSP^141^) [105,108,110,111,112] and inflammatory processes (VIM^56^ and NDRG^1330^) [107,113]. Both oxidative stress and inflammation are hallmarks of TiO_2_-induced cellular stress [1]. We show here that dysregulated phosphorylation of some key proteins may at least partly drive these effects.

## 4. Conclusions

Analysis of the phosphoproteome of A549 cells exposed to TiO_2_-NPs reveals that these nanoparticles affect DNA damage response, by means of dysregulation of the cell cycle and DNA repair, the process of autophagy, the cytoskeleton dynamics and structure, the RNA dynamics, as well as intracellular transport. It highlights the central role of p53 in some of these processes. Although these effects of TiO_2_-NPs have already been reported using other techniques, here we suggest that there is a link between the dysregulation of phophoprotein phosphorylation status and these cellular events, using an exposure condition that does not induce any overt cytotoxicity. It should be highlighted that the A549 cell line is derived from an alveolar carcinoma. Therefore, it would be important to repeat this experiment on a non-cancerous cell line. This would demonstrate that the observed effects are not specific to cancer cells and that they are representative of the impact caused by inhalation exposure of healthy individuals to TiO_2_-NP.

## Figures and Tables

**Table 1:** List of GO terms deduced from analysis of the list of phosphopeptides, with cutoff FDR ^1^ at 20%.

Category	Term	Count	%	*p*-Val.	List Tot.	Fold Enrich.	FDR
UP_KEYWORDS	Phosphoprotein	81	96.429	0.000	84	2.41	0.00
UP_KEYWORDS	Acetylation	40	47.619	0.000	84	2.86	0.00
UP_KEYWORDS	Cytoplasm	43	51.190	0.000	84	2.19	0.00
GOTERM_CC_DIRECT	cell-cell adherens junction	12	14.286	0.000	82	8.26	0.00
GOTERM_CC_DIRECT	nucleoplasm	32	38.095	0.000	82	2.55	0.00
UP_KEYWORDS	Methylation	18	21.429	0.000	84	4.41	0.00
GOTERM_BP_DIRECT	cell-cell adhesion	11	13.095	0.000	79	8.63	0.00
UP_KEYWORDS	Ubl conjugation	23	27.381	0.000	84	3.31	0.00
GOTERM_MF_DIRECT	protein binding	65	77.381	0.000	83	1.50	0.00
GOTERM_CC_DIRECT	focal adhesion	12	14.286	0.000	82	6.82	0.00
GOTERM_MF_DIRECT	cadherin binding involved in cell-cell adhesion	11	13.095	0.000	83	7.71	0.00
GOTERM_CC_DIRECT	cytosol	34	40.476	0.000	82	2.28	0.00
UP_KEYWORDS	Isopeptide bond	18	21.429	0.000	84	3.90	0.00
GOTERM_CC_DIRECT	cytoplasm	42	50.000	0.000	82	1.79	0.03
GOTERM_MF_DIRECT	poly(A) RNA binding	18	21.429	0.000	83	3.24	0.03
UP_KEYWORDS	Cytoskeleton	16	19.048	0.000	84	3.44	0.05
UP_KEYWORDS	Nucleus	39	46.429	0.000	84	1.82	0.06
UP_KEYWORDS	Alternative splicing	60	71.429	0.000	84	1.39	0.25
GOTERM_CC_DIRECT	actin cytoskeleton	7	8.333	0.000	82	7.14	0.52
GOTERM_CC_DIRECT	nucleus	39	46.429	0.001	82	1.60	0.98
UP_KEYWORDS	Host-virus interaction	8	9.524	0.001	84	5.09	1.13
GOTERM_CC_DIRECT	membrane	21	25.000	0.001	82	2.12	1.55
UP_SEQ_FEATURE	mutagenesis site	20	23.810	0.001	83	2.21	1.57
UP_KEYWORDS	Cell junction	10	11.905	0.002	84	3.63	1.90
GOTERM_BP_DIRECT	osteoblast differentiation	5	5.952	0.001	79	10.22	2.03
GOTERM_CC_DIRECT	Z disc	5	5.952	0.002	82	9.42	2.33
GOTERM_MF_DIRECT	actin binding	7	8.333	0.002	83	5.12	2.89
UP_SEQ_FEATURE	Nuclear localization signal	7	8.333	0.003	83	4.92	3.96
UP_KEYWORDS	Coiled coil	23	27.381	0.004	84	1.86	4.43
GOTERM_CC_DIRECT	cell projection	4	4.762	0.005	82	11.85	5.50
GOTERM_BP_DIRECT	microtubule cytoskeleton organization	4	4.762	0.004	79	11.98	6.27
UP_KEYWORDS	Apoptosis	8	9.524	0.006	84	3.66	6.95
GOTERM_BP_DIRECT	regulation of cellular response to heat	4	4.762	0.005	79	11.34	7.28
GOTERM_MF_DIRECT	14-3-3 protein binding	3	3.571	0.006	83	25.42	7.31
GOTERM_CC_DIRECT	perinuclear region of cytoplasm	9	10.714	0.006	82	3.22	7.55
GOTERM_BP_DIRECT	insulin receptor signaling pathway	4	4.762	0.006	79	10.90	8.09
GOTERM_CC_DIRECT	intracellular membrane-bounded organelle	8	9.524	0.012	82	3.19	13.93
INTERPRO	Microtubule associated protein 1	2	2.381	0.013	80	154.66	15.24
GOTERM_BP_DIRECT	viral process	6	7.143	0.013	79	4.27	17.15
GOTERM_BP_DIRECT	negative regulation of extrinsic apoptotic signaling pathway	3	3.571	0.013	79	16.78	18.06
UP_KEYWORDS	RNA-binding	8	9.524	0.018	84	2.95	19.65
GOTERM_BP_DIRECT	erythrocyte differentiation	3	3.571	0.015	79	15.94	19.73
KEGG_PATHWAY	Focal adhesion	5	5.952	0.020	36	4.64	19.96
UP_KEYWORDS	Fatty acid biosynthesis	3	3.571	0.019	84	14.14	20.54
GOTERM_CC_DIRECT	cytoplasmic vesicle membrane	4	4.762	0.019	82	7.06	20.83
GOTERM_MF_DIRECT	1-phosphatidylinositol-3-kinase activity	3	3.571	0.019	83	14.19	20.99

^1^ FDR: false discovery rate (in %) only FDR < 50% are reported. “List tot.”: number of input proteins retained for testing the GO term. “Count”: number of genes annotated with tested GO term. %: ratio between the two. *p*-Val. (*p*-value): statistical risk associated with tested GO term. FDR: false discovery rate taking into account multiple testing of GO terms. Fold Enrich (Fold Enrichment): composite probability score of the relevance of GO term.

**Table 2:** Dysregulated phosphorylation sites with a known biological function.

P-site	FC	Protein Activities	Site-Specific Phosphorylation Role	Kinase	Reference
KIF4A^801^	3	Kinesin motor, plays a role in mitosis	Inhibition of PRC1-microtubule overlaps at the central spindle during mitosis	AURORA-B	[101]
ERF^526^	2.25	Repressor of the transcription. Tumor suppressor. Sensor of ERK activity, affects cell cycle progression.	Regulates nuclear export of ERF. Promotes cell cycle arrest in G1 phase. Decreases ERF repression of the transcription	ERK	[99]
MISP^397^	2	Plays a role in mitosis: mitotic spindle orientation and mitosis progression.	Regulates mitotic spindle positioning, which is required for the definition of the correct cell division axis	PLK1	[104]
NDRG1^330^	1.7	Plays roles in apoptosis, cell growth and differentiation, mitosis, cell trafficking, hormone response. Tumor metastasis suppressor.	Cell cycle-dependent phosphorylation (temporally and spatially). Regulates the expression of CXC chemokine and acts on NFκB signaling pathway.	SGK1	[113]
VIM^56^	1.6	Transport of intracellular vesicles (lysosomes, secretion granules)	During acute inflammation, plays a role in granule secretion in neutrophils.	CDK5	[107]
FLNA^1084^	1.6	Links actin filaments to cell membrane glycoproteins, and various proteins to the actin cytoskeleton. Role in cell-cell contact and signal transduction	Involved in the separation of daughter cells after mitosis, as well as cell migration.	CDK1	[103]
ENSA^67^	1.5	Inhibitor of PP2A phosphatase	Regulates the activity of PP2A phosphatase, which regulates cyclin-B1-CDK1 activity high during M phase	CDK1-activated GWL	[100]
RB1^826^	1.5	Tumor suppressor. Regulator of cell division. Maintenance of heterochromatin structure	Regulate the binding of RB1 with E2F transcription factors, necessary for G1-S transition during cell cycle progression	CDK2-CyclinE/A	[102]
CARHSP1^41^	−1.6	Regulates the stability of mRNAs	Promotes/inhibits single-strand DNA binding. Localizes CRHSP1 at stress granules in condition of oxidative stress	DYRK isoform, among other kinase(s) of proline	[110,111,112]
PIK3C2A^259^	–2	Role in intracellular trafficking, particularly for insulin signaling, endocytosis process. Role in EGF signalling cascade, mitosis, response to UV-induced damage.	Phosphorylation regulates PIK3C2A conformation, its cellular localization and its degradation	CDC2 or UV irradiation+ JNK/SAPK	[114,115,116]
AKAP1^151^	−2	Regulates the localization of regulatory subunits of protein kinase A, either on mitochondrial membranes or in the endoplasmic reticulum.	AKA1 is associated with PP1 in G1 phase of the cell cycle; phosphorylation of S151 in AKAP1 leads to dissociation of this interaction at the G1/S transition.	PKC	[98]
PTGES3^113^	−2.1	Prostaglandin PGE2 synthase, promotes disassembly of transcription regulation complexes by binding on genomic response elements.	Phosphorylation activates this enzyme, by promoting the formation of a cPGES-CK-II-Hsp90 complex.	CK-II	[117]
HSPB1^15^	−2.3	Molecular chaperone. Protects unfolded proteins during oxidative stress. Role in actin filament dynamics, important for cell motility	Phosphorylated in response to cellular stress. Causes dissociation of Hsp complexes, which decreases their chaperone activity	MAPKAP2	[105,108]
CTTN^421^	−2.3	Involved in the organization of actin cytoskeleton. Role in cell migration, in the formation of metastases, in the formation of protein complexes at focal adhesions, in endocytosis and intracellular transport	Phosphorylation by SRC promotes cell motility, where phosphorylated CTTN acts as a linker between F-actin and focal adhesions. Promotes the endothelial cell barrier function enhancement triggered by S1P.	SRC in response to EGFR activation	[97,106,109]

FC: phosphorylation level fold-change, exposed cells vs. control cells.

## Supplementary Materials

The following are available online at https://www.mdpi.com/2079-4991/10/2/185/s1. Figure S1: Illustration of the three whole protein phosphorylation level indicators. Figure S2: Overall phosphorylation levels. Figure S3: analysis of the cell cycle. Table S1: List of MS/MS spectra assigned to human peptides in control cell datasets (Ctrl) and TiO2-NP-exposed cell datasets (TiO2). Table S2: List of unique phosphorylation sites detected in control cell datasets (Ctrl) and TiO2-NP-exposed cell datasets (TiO2). Table S3: List of detected phosphoproteins, provided together with phosphorylation counts. Table S4: List of GO terms identified using David with phosphopeptides. Table S5: List of GO terms identified using David with phosphocounts (clustered). Table S6: List of GO terms identified using David with P-sites. Tables S7–S9: List of GO terms (IPA) revealed from phosphoproteins from Cat. 1, Cat. 2 and Cat. 3, respectively.
