# Supplementary material for: Titanium Dioxide Nanoparticles Alter the Cellular Phosphoproteome in A549 Cells
Source: Nanomaterials (Basel). 2020 Jan 21;10(2):185. doi: 10.3390/nano10020185 (PMC7074930; doi:10.3390/nano10020185)
Supplement: Supplementary file 1 [file nanomaterials-10-00185-s001.zip › nanomaterials-685198/Supplementary_Material_corrected.docx]

Supplementary Materials for:

Titanium Dioxide Nanoparticles Alter the Cellular Phosphoproteome in A549 Cells

Mathilde Biola-Clier ^1^, Jean-Charles Gaillard ^2^, Thierry Rabilloud ^3^, Jean Armengaud ^2,^* and Marie Carriere ^1,^*

^1^ Univ. Grenoble-Alpes, IRIG, SyMMES, CIBEST, F-38000 Grenoble, France; mathilde.clier@gmail.com

^2^ Laboratoire Innovations technologiques pour la Détection et le Diagnostic (Li2D), Service de Pharmacologie et Immunoanalyse (SPI), CEA, INRA, F-30207 Bagnols-sur-Cèze, France;
jean-charles.gaillard@cea.fr

^3^ Chemistry and Biology of Metals, Univ. Grenoble Alpes, CNRS UMR5249, CEA, IRIG-DIESE-LCBM-ProMD, F-38054 Grenoble, France; thierry.rabilloud@cnrs.fr

***** Correspondence: jean.armengaud@cea.fr (J.A.); marie.carriere@cea.fr (M.C.)

1. Supplementary figures

**Figure S1.** Illustration of the three whole protein phosphorylation level indicators. This illustration takes the example of a twice-phosphorylated peptide detected three times: this peptide accounts for 6 according to the phosphorylation count, 3 with the phosphopeptide count and 2 according to the phosphorylated site count.


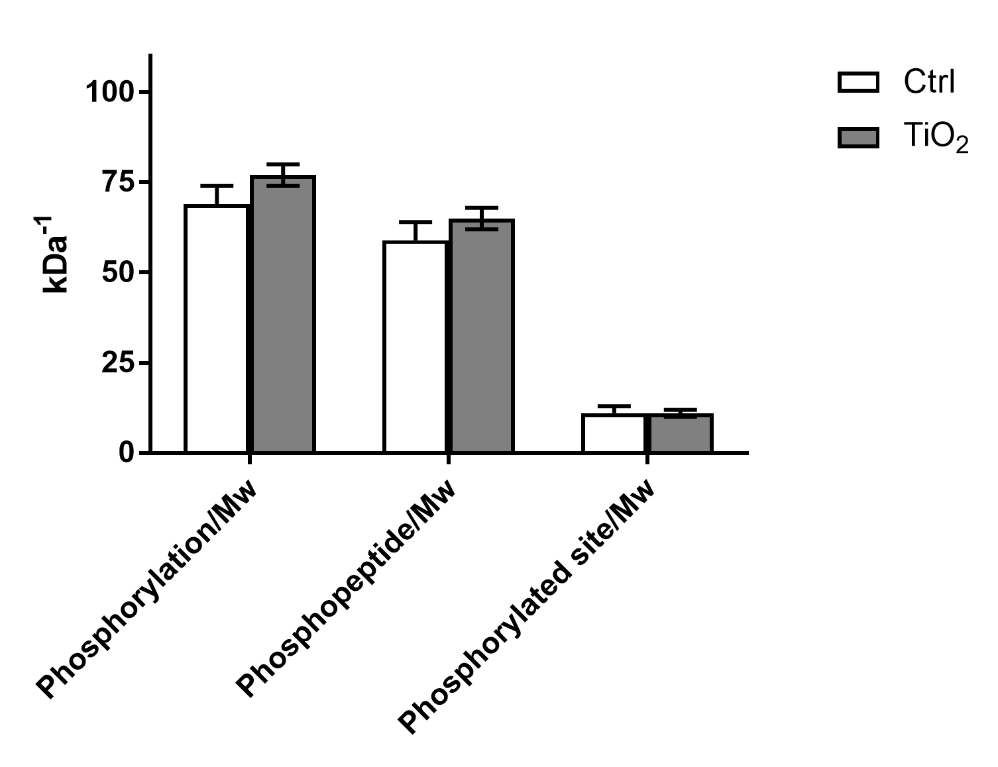


**Figure S2.** Overall phosphorylation levels. Overall phosphorylation levels of phosphoproteins detected in control cells (CTL) and cells exposed to 100 µg/ml TiO_2_-NP. Results are normalized with respect to individual protein molecular weights (Mw). Mean ± standard deviation, n=3; statistical significance (*): p<0.05, exposed vs. control (none of the conditions showed any significant difference).

**
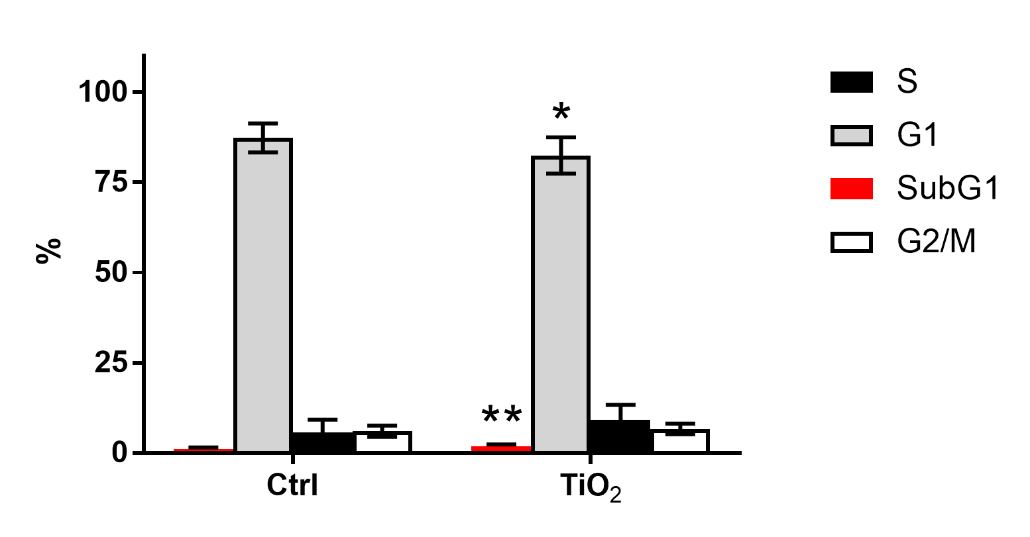
**

**Figure S3.** Cell cycle analysis. Cell cycle phase distribution of A549 cells unexposed cells (CTL) or cells exposed to 100 µg/ml TiO_2_ NPs for 24 h. Mean ± standard deviation, n=3; statistical significance, exposed vs. control (*): *p* < 0.05, (**): *p*<0.005.
